# Chronic Exposure to Ambient Ozone and Asthma Hospital Admissions among Children

**DOI:** 10.1289/ehp.11184

**Published:** 2008-09-10

**Authors:** Shao Lin, Xiu Liu, Linh H. Le, Syni-An Hwang

**Affiliations:** 1 Center for Environmental Health, New York State Department of Health, Troy, New York, USA; 2 Bureau of Healthcom Network System Management, New York State Department of Health, Menands, New York, USA

**Keywords:** air pollution, asthma, children, chronic, hospital admissions, ozone

## Abstract

**Background:**

The association between chronic exposure to air pollution and adverse health outcomes has not been well studied.

**Objective:**

This project investigated the impact of chronic exposure to high ozone levels on childhood asthma admissions in New York State.

**Methods:**

We followed a birth cohort born in New York State during 1995–1999 to first asthma admission or until 31 December 2000. We identified births and asthma admissions through the New York State Integrated Child Health Information System and linked these data with ambient ozone data (8-hr maximum) from the New York State Department of Environmental Conservation. We defined chronic ozone exposure using three indicators: mean concentration during the follow-up period, mean concentration during the ozone season, and proportion of follow-up days with ozone levels > 70 ppb. We performed logistic regression analysis to adjust for child’s age, sex, birth weight, and gestational age; maternal race/ethnicity, age, education, insurance status, smoking during pregnancy, and poverty level; and geographic region, temperature, and copollutants.

**Results:**

Asthma admissions were significantly associated with increased ozone levels for all chronic exposure indicators (odds ratios, 1.16–1.68), with a positive dose–response relationship. We found stronger associations among younger children, low sociodemographic groups, and New York City residents as effect modifiers.

**Conclusion:**

Chronic exposure to ambient ozone may increase the risk of asthma admissions among children. Younger children and those in low socioeconomic groups have a greater risk of asthma than do other children at the same ozone level.

The acute health effects of exposure to ambient ozone have been examined in many geographic regions. Although the findings are inconsistent, potential adverse effects reported include decrements in lung function, airway inflammation, symptoms of asthma, increases in hospitalization for respiratory diseases, and excess mortality (Schwela 2000). On the other hand, the health effects of chronic exposure to ozone have been less studied, and the results are inconclusive. Several studies have reported that long-term exposure to ozone may reduce lung function in school children and adults (Galizia and Kinney 1999; Peters et al. 1999; Qian et al. 2005; Rojas-Martinez et al. 2007) and increase the prevalence of asthma and asthmatic symptoms (Hwang et al. 2005; Ramadour et al. 2000; Wang et al. 1999). In contrast, other studies found an insignificant or negative association between respiratory health effects and chronic ozone exposure (Gauderman et al. 2004; Karr et al. 2007; Zemp et al. 1999). No studies have examined health effects of chronic ozone exposure among a birth cohort. Biologic studies provided plausibility that chronic exposure to ozone is associated with elevated oxidative damage, which might be involved in development of asthma and decrement of lung function (Chen et al. 2007; Mudway and Kelly 2000).

In New York State, asthma hospitalization rates remain considerably higher (20.4 per 10,000) than the U.S. rate of 16.9 per 10,000 population in 2002 (American Lung Association 2004; New York State Department of Health 2005). Young children are a vulnerable population with respect to air pollutants, given their narrow airways, higher breathing rate, developing lungs and immune systems, and more time spent outdoors. Most prior studies have focused on adults and school-age children and analyzed less severe health outcomes than hospitalizations. The effect of chronic exposure to high levels of air pollution in early life and its subsequent impact on childhood asthma admissions is unclear. In addition, the interactive effects between maternal/birth characteristics and air pollution on childhood asthma hospital admissions have rarely been studied. The purpose of this study was to investigate the association between chronic exposure to ozone and childhood asthma admissions. We also examined whether maternal and birth characteristics interacted with the impact of air pollution on asthma hospitalization in early life.

## Materials and Methods

### Study population and health outcome

In this retrospective cohort study we analyzed a New York State birth cohort born between 1 January 1995 and 31 December 1999 and followed each individual until the first asthma hospital admission or 31 December 2000. We defined asthma hospital admissions as children who were first admitted to a hospital from 1 January 1996 through 31 December 2000 with a principal diagnosis of *International Classification of Diseases, 9th Revision*, code 493 (U.S. Department of Health and Human Services 1991). We excluded children residing in Staten Island from the study cohort because a substantial amount of data was missing for ambient ozone in that area. We did not consider admitted children who were < 1 year of age because of difficulties in differentiating asthma from bronchiolitis in this age group (Kramarz et al. 2000). This study was approved by the New York State Department of Health Institutional Review Board and complied with all applicable U.S. regulations.

We obtained information on hospital admissions and maternal and birth characteristics from the New York State Integrated Child Health Information System (ICHIS). The ICHIS links various sources of New York State data, including the immunization records, birth certificates, hospital discharges, and death certificates. The birth certificate portion included individual-level information on residential address, maternal demographics, and birth characteristics; the hospital discharge portion contained the cause and date of hospital admission and home address at admission. We obtained regional demographic information from the 2000 U.S. Census (U.S. Census Bureau 2000), including land area, population density, hospital density, percentage of individuals living below poverty level, and median household income. Thus, potentially important individual and neighborhood confounders can be controlled when assessing the relationship between childhood asthma admissions and chronic exposure to ambient ozone.

### Air pollution data and exposure indicators

**Figure 1:**
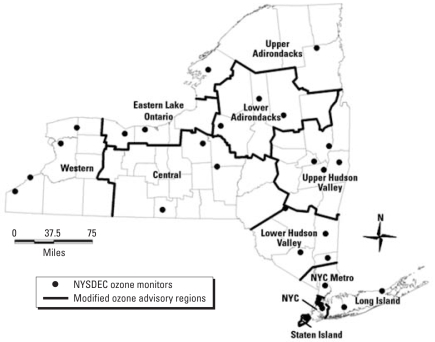
Ozone exposure regions in New York State used in this study. NYC, New York City.

We obtained hourly ambient ozone data from the New York State Department of Environmental Conservation (NYSDEC). These data (reported in parts per billion) were measured hourly for each day. We used the 8-hr maximum hourly value during the peak outdoor exposure time (1000 to 1800 hours) in this study to represent the daily ozone level. We included 32 ozone monitoring sites and divided them into 11 ozone regions in New York State (Figure 1) according to their spatial and temporal correlations, wind direction, and traffic pattern. In general, we used a linear regression model with a fixed-origin intercept to assess the spatial correlation among monitor pairs as a function of distance. We performed empirical semivariance analysis and a total count of occurrences above a specific concentration to evaluate the spatial variation. The resulting 11 ozone regions are very similar to the air quality health advisory regions developed by NYSDEC (2008). If more than one monitoring site was located in one ozone region, we used the average value of the 8-hr maximum from multiple monitoring sites to represent the regional data. We assessed children’s chronic ozone exposure as both continuous and categorical variables by identifying the individual ozone region based on their residential histories. The continuous ozone variable included the mean concentrations and the exceedance proportion. Mean concentration was the arithmetic mean of the regional 8-hr ozone maximum for each child during two different time periods: the entire follow-up period and ozone seasons (April–October) during the follow-up period. For children who had hospital admissions during the defined periods, we calculated the mean concentration between the date of birth and the date of admission. For children who had no hospital admissions, we calculated the mean concentration of the follow-up period and the ozone season between the date of birth and the end of the study. We assessed the risk of hospital admissions associated with chronic ozone exposure using a 1-ppb increase in mean concentration during the defined period. The exceedance proportion is the proportion of follow-up days with ozone levels > 70 ppb, which is our 90th percentile of year-round ozone over the entire follow-up period. This indicator not only reflects the specific days exposed to high ozone levels, but also includes the proportion of days exposed to high ozone levels for each child over the follow-up period. Therefore, it captures both the cumulative and chronic nature of exposures. We used the interquartile range (IQR; 75th to 25th quartile) as the unit change in the models for the continuous exceedance proportion exposure.

To estimate the potential dose–response effect, we categorized children as having low, medium, or high ozone exposure based on the tertile ranking of their mean ozone concentration during the follow-up period in New York City and the rest of New York State separately to adjust for geographic differences. The ranges for low, medium, and high exposure were 31.46–37.29 ppb, 37.30–38.11 ppb, and 38.12–50.13 ppb, respectively, for children living in New York City; and 33.50–42.57 ppb, 42.58–45.06 ppb, and 45.07–55.19 ppb for children living in other New York State regions.

### Data linkage methods and geocoding

According to the enrollment criteria described above, we identified 1,433,826 children from the ICHIS database. We geocoded residential addresses at birth (from the birth certificate database) at the street level to determine the geographic region of air pollution using Map Marker (Mapinfo Corp., Troy, NY). In general, we geocoded 84% of the residential addresses automatically; we could not geocode 16% and excluded them from analysis. To assign an ozone region to individual residential addresses, we overlaid the map of geocoded addresses onto the map of ozone regions using Mapinfo (Mapinfo Corp.). Finally, we linked the ambient ozone data from NYSDEC to the asthma outcome, individual risk factors from ICHIS data, and regional level factors from the U.S. Census (U.S. Census Bureau 2000).

To evaluate study population dynamics, we compared exposure regions based on the original residential addresses retrieved from the birth certificate database to the secondary residences extracted from the immunization record and hospital discharge databases. Our study population was very stable, and we found a consistent ozone region (i.e., change in address did not affect exposure region classification) for 72.6% of children. For 27.2% of children, address information could be obtained from only one source: 18.9% from the birth certificate only and 8.3% from the immunization record only. For these children, we geocoded the residential address from the unique database. Only 0.2% of children had address changes that placed them in a different ozone region. For these children, we calculated the effective exposure corresponding to the time window across these regions. If a child was born in ozone region “A” and was admitted to the hospital at a later date in region “B,” we considered the child spent half the time in region “A” and half the time in region “B.”

### Statistical analysis

In this study we aimed to predict the risk of having asthma admissions in a birth cohort, but the time to the first admission in children that is usually analyzed in survival models was not our primary interest. We therefore used a multivariate logistic regression model rather than a survival analysis. By incorporating children’s ages (at admission or the end of the study) in the logistic regression analysis, we also appropriately considered and controlled the time dimension of exposure. We computed adjusted odds ratios (ORs) and 95% confidence intervals (CIs) while controlling for birth and maternal confounding variables and geographic regions. We fitted regression models separately for continuous and categorical chronic ozone exposures. We selected confounding variables based on the epidemiologic literature and the exploratory results using univariate analyses that suggested possible associations with asthma admissions at *p*-values ≤0.05. These confounders included child’s sex, birth weight (≤ 2,500 g, > 2,500 g), gestational age (< 260 days, ≥ 260 days), age at admission or end of the study (range, 1–6 years) maternal age at delivery (< 20 or > 35 years, 20–35 years) and smoking status during pregnancy (yes, no). As a measure of sociodemographic status, we controlled for individual-level risk factors such as maternal race (black, other), ethnicity (Hispanic, non-Hispanic), education level (< 12 years, ≥ 12 years), insurance type during pregnancy (Medicaid, self-paid, and other) and geographic area (New York City vs. other). We also ascertained census block–group information such as median household income, percentage of population living below poverty level (the highest quartile vs. others), and hospital density (i.e., number of hospitals per 100 km^2^ in each ozone region) to control for medical care access at the neighborhood level. Because hospital density was highly correlated with geographic region (Pearson correlation *r* = 0.99), and median household income was correlated with percent below poverty (Pearson correlation *r* = 0.69), we retained all confounders except for hospital density and median household income in the final multivariate regression models because of significant *p*-values (< 0.01). We also adjusted the proportion of days during the entire follow-up period with extreme temperatures, defined as the 90th percentile of the daily average temperature (72.3°F), in the final model, in which we compared children in the highest quartile of extreme temperature days (high-temperature group) with all others (low-temperature group).

We also assessed and controlled for the effects of copollutants by using the Air Quality Index (AQI) (U.S. Environmental Protection Agency 1999), which is a standard system representing the levels of multiple air pollutants, including ambient particulate matter (particulate matter with aerodynamic diameters ≤ 10 and 2.5 μm), nitrogen dioxide, carbon monoxide, and sulfur dioxide. We calculated the AQI for each pollutant in each ozone region and chose the maximum value to represent the daily AQI for that region. Cumulative AQI was the average level of daily AQI during the follow-up period. We used the AQI variable only in the analysis for children born between 1996 and 1999, but did not retain it in the final analysis model because AQI data were not available for 1995 and we would have missed 250,000 eligible births if we had included AQI.

We further added interaction terms to the logistic regression model with continuous ozone exposure to assess potential effect modifications. We used mean ozone concentration during the entire follow-up period to create the interaction terms with all dichotomous confounders. Once we identified significant interaction terms (*p*-value < 0.05), we performed stratified analysis to assess the differential impacts of birth and maternal risk on the association between air pollution and asthma admissions. The Hosmer–Lemeshow test showed that the models fitted reasonably well. We performed all statistical analyses with SAS software, version 9.1 (SAS Institute, Inc., Cary, NC).

## Results

We included 1,204,396 eligible births in the analysis, with 10,429 (0.87%) children admitted to the hospital for asthma by 31 December 2000. Children in this study were between 1 and 6 years of age. Overall, the average follow-up time was 26.6 months for children with asthma admissions and 42 months for all births. The mean ozone level during the entire follow-up period (41.06 ppb) was lower than the mean concentration in the ozone season (50.62 ppb) (data not shown). The average exceedance proportion was 9.72% among the study population, ranging from 1.66% to 26.27%. Table 1 summarizes the average ozone levels, total area of region, and key population characteristics by exposure regions. The western region had the highest ozone level in New York State (47.8 ppb), and New York City had the lowest ozone level among 10 New York State regions (37.5 ppb). However, population density, birth density, percentages of blacks and Hispanics, and low level of maternal education in New York City were significantly higher than in the rest of New York State.

As described in Table 2, we found significant positive associations between chronic ozone level and asthma hospital admissions for all exposure indicators after adjusting for potential confounding variables (ORs = 1.16–1.68). The risk of hospital admissions increased 22% with a 1-ppb increase in mean ozone concentration during the ozone season. Indicators using the entire follow-up period (OR = 1.16) showed similar but weaker elevated risks for asthma admissions. When estimating the ozone effect using the exceedance proportion, we observed a significant increase (OR = 1.68; 95% CI, 1.64–1.73) in hospital admissions associated with an IQR (2.51%) increase in ozone.

Because the effects of other risk factors on asthma admissions were similar when using three ozone indicators, to simplify the results Table 2 shows the effects of other risk factors using only the mean concentration during the entire follow-up period as the exposure included in the model. Children who are black or Hispanic, whose mother had a lower level of education, Medicaid, or self-paid insurance, and those residing in areas with a high percentage of below-poverty populations had a higher risk of asthma admissions. Low birth weight, preterm birth, and maternal smoking during pregnancy were associated with increased asthma admissions. Geographic region was the strongest risk factor associated with asthma admissions in this study. Children living in New York City were 4.21 (95% CI, 3.77–4.70) times more likely to be admitted to a hospital than children living in other regions of New York State. Child’s age and female sex were negatively associated with asthma admissions. Maternal age and extreme temperature were not associated with increased asthma admissions. Additionally, we also examined the ozone–asthma association during the entire follow-up period after controlling for AQI and other confounders. The adjusted OR after controlling for AQI increased slightly (data not shown; OR = 1.24; 95% CI, 1.23–1.25) compared with the unadjusted OR of 1.16.

To control for the impacts of other confounders such as admission policy, home environment, and disease management, which are unavailable in ICHIS data but are related to hospital admissions, we used a “negative control,” admissions due to gastroenteritis, in a sensitivity analysis. We found that chronic exposure to ozone was not positively associated with hospital admissions due to gastro-enteritis (OR = 0.57; 95% CI, 0.54–0.59) after adjusting for confounders similar to those used in the asthma analysis.

**Figure 2:**
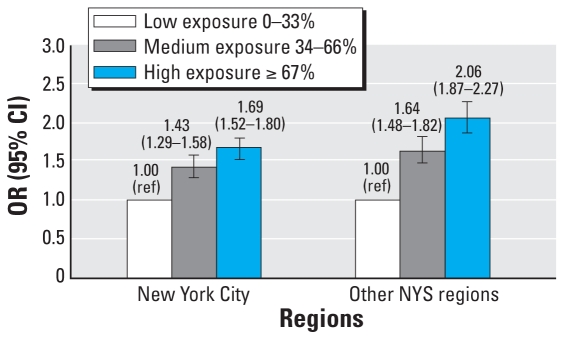
Ozone–asthma dose–response relationship using the mean concentration during the entire follow-up period, adjusted for child’s sex, age, birth weight, and gestational age; maternal race, ethnicity, age, education, insurance, and smoking status during pregnancy; and regional poverty level and temperature. NYS, New York State. ref, referent.

To address the relationship between ozone dose and health effects, we analyzed dose response separately with categorical exposures in New York City and other regions of New York State (Figure 2). After adjustment for other covariates, we observed significantly positive dose–response relationships in both regions; that is, higher ozone exposure was associated with a higher risk of admissions.

**Figure 3:**
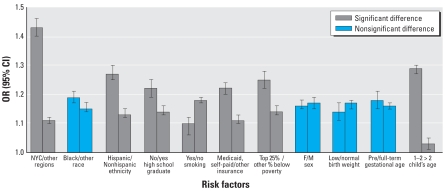
Stratified analysis of ozone–asthma association, using the mean concentration during the entire follow-up period. NYC, New York City.

We first identified the interactive effect of maternal/infant factors and chronic ozone levels on asthma hospital admissions from the logistic regression model and then investigated it using stratification analysis (Figure 3). We found the largest disparity for geographic regions; that is, the ozone-asthma association was stronger among New York City residents (OR = 1.43) than among other regions (OR = 1.11). Similarly, the effects of ozone on asthma admission were significantly higher among children 1–2 years of age (OR = 1.29), children living in poor neighborhoods (i.e., highest quartile of percent below poverty; OR = 1.25), children whose mothers had low education (OR = 1.22), Medicaid/self-paid births (OR = 1.22), and Hispanics (OR = 1.27) compared with older children (OR = 1.03), children in good neighborhoods (OR = 1.14), mothers with high school education (OR = 1.14), births covered by other insurance (OR = 1.11), and non-Hispanic children (OR = 1.13). However, we found the ozone–asthma association to be weaker among children born to smokers than among those born to nonsmokers (OR = 1.10 and 1.18, respectively).

## Discussion

In this study, we found that chronic exposure to ambient ozone in early life was significantly and positively associated with an increased risk of asthma hospital admissions among a birth cohort in New York State. Multivariate analyses using three different exposure indicators produced similar results. We also observed positive dose–response trends in New York City and the remainder of New York State.

Many time-series studies have found that hospitalizations or emergency visits due to asthma may increase during the periods of short-term exposure to increased ozone levels (Burnett et al. 2001; Gent et al. 2003; Tolbert et al. 2000). Although few other studies have assessed the risk of childhood asthma hospitalizations associated with chronic ozone exposure, the findings of this study are consistent with several previous studies among children. Three cross-sectional studies observed a positive relationship between chronic exposure to ozone, and prevalence of asthma and asthmatic symptoms in school children (Hwang et al. 2005; Ramadour et al. 2000; Wang et al. 1999). Peters et al. (1999) and Rojas-Martinez et al. (2007) have reported a reduction in lung function associated with higher ozone concentration among school-age children in two prospective studies. One cohort study in Southern California found that in communities with high ozone levels, children who played sports outdoors had 3.3 (95% CI, 1.9–5.8) times the risk of developing asthma compared with children not playing sports (McConnell et al. 2002). Another birth cohort study by Brauer et al. (2007) also reported a significant association (OR = 1.26) between traffic-related pollution and both doctor-diagnosed asthma and self-reported wheeze among children < 4 years of age, but ozone was not measured in that project. No previous studies examined a similar health effect from chronic ozone exposure among young children in a birth cohort for direct comparison with our study. Taken together with the present study, all study findings in the current literature indicate that ozone might have both acute and chronic effects on the development of asthma and cause a subsequent increase in asthma hospital admissions. On the other hand, other studies (Gauderman et al. 2004; Zemp et al. 1999) found no significant association between exposure to ozone and respiratory morbidity, and Karr et al. (2007) reported a reduced risk (OR = 0.92; 95% CI, 0.88–0.97) of hospitalization for infant bronchiolitis with a 14-ppb increase in ozone exposure. Despite these studies showing no effect, our study has contributed another confirmation of positive associations between ozone exposure and respiratory outcomes.

Interestingly, in the present study, the associations between elevated ozone and increased asthma hospital admissions were stronger in young children, Medicaid/self-paid births, Hispanics, children living in poor communities or whose mothers had low education levels, and New York City residents. These results suggest that chronic exposure to ambient ozone did not have an equal asthma effect on children. Specifically, very young children were more susceptible to ozone exposure, and children with low socioeconomic status (SES) had a greater risk of asthma admissions at the same ozone level. Children < 2 years of age have been reported to be more susceptible to air pollutants compared with other children (Braga et al. 2001). Son et al. (2006) found a similar interactive effect of SES characteristics on the relationship between short-term exposure to high ozone levels and asthma hospital admissions. In New York State, Lin et al. (1999) found that areas with a high proportion of low-income, low-education level and high population density (i.e., crowded living conditions) experience higher rates of asthma hospitalizations. In the present study, maternal smoking status during pregnancy weakened the ozone effect on asthma admissions. This finding has not been reported in previous air pollution studies and should be interpreted with caution because maternal smoking information from the birth certificate was based on self-report. Smoking tends to be underreported by study participants in general, and smoking while pregnant is stigmatized. In addition, some quitters might go back to smoking after their children were born or some time before the child’s hospital admission.

Although survival analysis is a method commonly used to examine time-varying events, logistic regression analysis has also been widely used in many air pollution and environmental epidemiologic studies (Andersen et al. 2008; Brauer et al. 2007; Hwang et al. 2005; Kumar et al. 2008; Morgenstern et al. 2008; Ramadour et al. 2000; Wang et al. 1999). Because the purpose of the present study is to predict the probability of occurrence of a hospital admission for asthma rather than time to disease occurrence, logistic regression analysis is an appropriate option. Additionally, in a survival analysis of a birth cohort, time to event is directly correlated with child’s age. However, asthma hospital admissions are also strongly associated with age. Therefore, by including child’s age in our logistic regression model, we could not only control for the confounding (and/or independent) effect of age but also incorporate the varying time dimension of exposure—that is, the length of time exposed for each child—which is the same as child’s age. To validate our analytical method and the findings, we conducted a proportional hazards model for the same data in New York City. This sensitivity analysis yielded similar results between asthma admissions and chronic exposure to ozone.

This is one of the largest studies (*n* = 1,204,396 births) to date examining the relationship between chronic exposure to ambient ozone during early life and asthma hospital admissions among children. Data obtained from ICHIS had health, birth, and maternal information and were then combined with air pollution data. This integrated environmental health database provides a unique birth cohort to follow health end points and to estimate incidence. We selected a vulnerable subgroup, children < 6 years of age, as our study population. Unlike adults, children may be more affected by air pollutants because of higher vulnerability and different behavior (Trasande and Thurston 2005). Children’s lungs and immune systems are developing, and exposure to high ozone levels may impair the normal process of development (Plopper and Fanucchi 2000; Trasande and Thurston 2005). In addition, children are more active than adults are and tend to be outside in the summer and the afternoon, which may result in higher individual exposure to ozone (Trasande and Thurston 2005; U.S. Environmental Protection Agency 2002). This study also used asthma hospital admissions from an existing file as the health outcome, which comes from the hospitals and is not subject to self-reporting bias. Although some studies have used cumulative ozone as an exposure indicator, to our knowledge this is the first study to specifically examine the association between chronic exposure to ambient ozone and first asthma hospital admission in children. This study also assessed the interactive effects of maternal/infant factors on the ozone–asthma association, the confounding effects of temperature and AQI, and the ozone effect on a control disease to estimate confounding bias.

Several limitations should be considered when interpreting these findings. First, hospital admissions data capture only the most severe cases of asthma and may not be representative of mild or less severe cases. Second, we have no personal activity information, so we are unable to calculate individual cumulative exposure accurately. Third, although many important confounding variables have been controlled in the analysis, further adjustment of other confounders such as genetic susceptibility may alter the associations. Finally, ICHIS data can identify the address changes over time only when a hospital admission occurred or if immunization data are available. For children who have a residential address from only one database, the accuracy of their exposure calculation is questionable. However, this misclassification bias would be nondifferential and toward the null. Our data also suggested stability in the study population, because most of the population did not change their exposure region over time, and few children lived in multiple ozone regions.

This study provides evidence of a positive association between chronic exposure to ozone and childhood asthma hospital admissions. Children who are exposed to high ozone levels over time are more likely to develop asthma severe enough to be admitted to the hospital. Further adjustment for temperature, AQI, and maternal/birth factors did not alter the ozone–asthma association. This study also found that socioeconomic characteristics may contribute to differential ozone–asthma associations and should be of special concern. The findings from the present study not only will help health professionals identify environmental risk factors for asthma admission, but also will help discern the impairment from chronic exposure to high ozone levels over time. Minimizing outdoor exposure opportunities on high-ozone days may be a viable strategy for reducing the burden of asthma hospital admissions in children. Further studies in a birth cohort examining health effect of chronic ozone exposure are needed to confirm our findings.

## Figures and Tables

**Table 1:** Summary of ozone levels, land use, and population characteristics by ozone region in New York State.

	Mean ozone^a^ (ppb) during follow-up	Land area^b^ (km^2^)	Population density^b^ (per km^2^)	Birth density in cohort (per km^2^)	Percent black in cohort	Percent Hispanic in cohort	Percent maternal low education in cohort
Ozone region							
Upper Adirondacks	44.45	21,820	18.0	0.20	3.46	3.20	15.52
Lower Adirondacks	43.86	16,806	23.5	0.26	4.70	2.67	17.27
Central	45.38	30,414	52.6	0.60	7.08	2.36	16.58
Upper Hudson Valley	46.21	15,118	73.0	0.81	7.97	4.17	14.15
Lower Hudson Valley	44.74	10,218	94.8	1.16	8.18	11.95	15.09
Western	47.78	16,699	95.3	1.15	12.96	4.11	14.47
Eastern Lake Ontario	41.84	5,741	165.7	2.16	16.33	6.87	16.64
New York City metro^c^	40.49	1,572	769.7	10.70	15.57	24.51	12.66
Long Island	42.69	3,105	886.9	11.76	10.15	16.14	10.49
New York City^d^	37.51	634	11929.4	174.76	34.32	41.27	25.49

a The average of the maximum hourly concentration measured between 1000 and 1800 hours during the follow-up period (1 January 1995 to 31 December 2000).

b We used 2000 census data (U.S. Census 2000) to obtain land area and population density information.

c Includes Rockland and Westchester Counties.

d Includes Bronx, New York, Kings, and Queens Counties.

**Table 2:** Association between ozone exposure indicators, birth and maternal risk factors, and asthma hospitalizations.

Characteristic	Adjusted OR (95% CI)
Ozone exposure (1-ppb increase/day)^a^	
Mean concentration during the follow-up period	1.16 (1.15–1.17)
Mean concentration during the ozone season	1.22 (1.21–1.23)
Exceedance proportion (%) > 70 ppb with IQR increase^b^	1.68 (1.64–1.73)
Other risk factors^c^	
Temperature	1.06 (1.00–1.13)
Child’s age (month)	0.93 (0.93–0.94)
Race: black vs. other	1.97 (1.88–2.07)
Sex: female vs. male	0.58 (0.56–0.61)
Ethnicity: Hispanic vs. non-Hispanic	1.99 (1.89–2.09)
Birth weight: low vs. normal	1.55 (1.44–1.67)
Gestational age: preterm vs. full term	1.36 (1.27–1.45)
Maternal education: < high school vs. ≥ high school	1.18 (1.12–1.24)
Maternal smoking during pregnancy: yes vs. no	1.39 (1.29–1.49)
Geographic region: New York City vs. other regions	4.21 (3.77–4.70)
Maternal age (years): < 20 or > 35 vs. 20–35	1.06 (1.00–1.11)
Poverty level: highest 25th quartile vs. other	1.21 (1.15–1.27)
Maternal insurance	
Medicaid vs. other	1.26 (1.19–1.33)
Self-paid vs. other	1.19 (1.10–1.29)

a Adjusted for geographic region, child’s sex, child’s age, birth weight, gestational age, maternal race, ethnicity, maternal age, education, maternal insurance, smoking status during pregnancy, poverty level, and temperature.

b IQR is a 2.51% increase.

c ORs are from the model including the mean concentration during the follow-up period.
